# Reach and public health implications of proposed new food marketing regulation in Germany: an updated analysis

**DOI:** 10.1093/eurpub/ckae087

**Published:** 2024-05-22

**Authors:** Anna Leibinger, Nicole Holliday, Oliver Huizinga, Carmen Klinger, Elochukwu Okanmelu, Karin Geffert, Eva Rehfuess, Peter von Philipsborn

**Affiliations:** Chair of Public Health and Health Services Research, Institute of Medical Information Processing, Biometry and Epidemiology (IBE), Faculty of Medicine, LMU Munich, Munich, Germany; Pettenkofer School of Public Health, Munich, Germany; Chair of Public Health and Health Services Research, Institute of Medical Information Processing, Biometry and Epidemiology (IBE), Faculty of Medicine, LMU Munich, Munich, Germany; Pettenkofer School of Public Health, Munich, Germany; German Non-Communicable Disease Alliance (DANK), Berlin, Germany; Chair of Public Health and Health Services Research, Institute of Medical Information Processing, Biometry and Epidemiology (IBE), Faculty of Medicine, LMU Munich, Munich, Germany; Pettenkofer School of Public Health, Munich, Germany; Chair of Public Health and Health Services Research, Institute of Medical Information Processing, Biometry and Epidemiology (IBE), Faculty of Medicine, LMU Munich, Munich, Germany; Pettenkofer School of Public Health, Munich, Germany; Chair of Public Health and Health Services Research, Institute of Medical Information Processing, Biometry and Epidemiology (IBE), Faculty of Medicine, LMU Munich, Munich, Germany; Pettenkofer School of Public Health, Munich, Germany; Chair of Public Health and Health Services Research, Institute of Medical Information Processing, Biometry and Epidemiology (IBE), Faculty of Medicine, LMU Munich, Munich, Germany; Pettenkofer School of Public Health, Munich, Germany; Chair of Public Health and Health Services Research, Institute of Medical Information Processing, Biometry and Epidemiology (IBE), Faculty of Medicine, LMU Munich, Munich, Germany; Pettenkofer School of Public Health, Munich, Germany

## Abstract

Advertising for unhealthy foods adversely affects children’s food preferences and intake. The German government published plans to restrict such advertising in February 2023 and has revised them several times since. We assess the reach of the current draft from June 2023, and discuss its public health implications. We show that across 22 product categories covered by the current draft law, the median share of products permitted for marketing to children stands at 55%, with an interquartile range of 11–73%. Resistance from industry groups and from within government poses hurdles and leaves the prospects of the legislation uncertain.

## Introduction

Exposure to marketing of unhealthy foods is considered a key contributing factor to unhealthy diets and diet-related chronic disease.^1^ In 2019, children in Germany using media were exposed, on average, to 20 food advertisements per day, 15 of which were for foods classified as unhealthy based on the 2015 edition of the Nutrient Profile Model of the World Health Organization Regional Office for Europe (WHO NPM).^2^ Against this backdrop, Germany’s Federal Ministry of Food and Agriculture published details on a planned Children's Food Advertising Act in February 2023.^3^ According to the proposed law, advertising to which children are exposed would be limited to products meeting the criteria of the 2023 edition of the WHO NPM. The legislation would cover most relevant advertising channels and use a comprehensive definition of exposure (see Panel 1 for details).^4^ 
 

Panel 1: Definition of exposure in the current draft (June 2023) of the Children's Food Advertising Act in Germany^3^

- **Exposure**: Any advertisement meeting at least one of the following three criteria would be deemed likely to be seen by a significant number of children, and would therefore be subject to the law:
  o Any advertisement whose content shows characteristics of being directed towards or appealing to children (e.g. by using child-like language, comic figures or children as protagonists).
  o Any advertisement shown in a context in which it is likely to reach an elevated number of children (e.g. during children’s programs on TV, or up to 100 m around schools and kindergartens).
  o Any TV advertisement aired between 5 and 10 pm on Mondays through Fridays, between 8 and 11 am and between 5 and 10 pm on Saturdays, and between 8 am and 10 pm on Sundays.
- **Channels**: The law would cover all relevant marketing channels (including TV, radio, print media, internet including social media and influencer marketing, outdoor advertisement and sponsoring).
- **Definition of children**: Children are defined as anyone younger than 14 years.

In the initial draft (February 2023), two adaptations to the original WHO NPM were included: The total sugar threshold for 100% fruit juice was removed as was the total fat threshold for dairy milk drinks.^5^ Following intense criticism from food and advertisement industry groups, and pressure from within government, further revisions were made. The total fat threshold for plant-based milks and for fresh and frozen meat, fish and eggs was removed, as were the total and saturated fat thresholds for yogurt and cream. Restrictions regarding advertising time and location have also been loosened (see Supplementary Material for details).^6^ In the present article, we analyze how these adaptations, reflected in the current draft (June 2023), affect the share of products permitted for marketing to children, updating an earlier analysis that was based on the initial draft of the Children's Food Advertising Act.^7^

## Methods

To assess the reach of the proposed law, we applied the revised nutrient and ingredient criteria of the law’s current draft to a dataset of randomly selected food and beverage products from the German market used in our earlier analysis of the law’s initial draft.^7^ Details on the sampling and the analytical procedures have been published elsewhere.^7^ In short, we randomly sampled food products on the German market from the open-source online database Open Food Facts. Subsequently, we followed the guidelines outlined in the WHO NPM manual to assign these to the 22 product categories specified by the WHO NPM. We randomly selected 30 products for each product category, 660 in total. We then calculated the share of products meeting all nutrient and ingredient criteria of the WHO NPM, the initial draft law and the current draft law. Besides, we defined several hypothetical reformulation scenarios, assuming reductions in sodium, sugar, total fat and energy by between 10 and 30%, and calculated the share of products permitted for marketing to children under these. All analyses were carried out in RStudio version 2023.06.1.

## Results

The median share of products meeting all nutrient and ingredient criteria of the law’s current draft and thus allowed for marketing to children, stands at 55% (IQR: 11–73%) across the 22 product categories covered by the law. This contrasts with a median share of 20% (IQR: 3–59%) under the original WHO NPM and a median share of 38% (IQR: 11–73%) under the initial draft law from February 2023 (see Table 1). Within specific product categories, the latest threshold adaptations raised the share of products that would be permitted for marketing to children from 70 to 73% in the category of plant-based milks, from 13 to 73% in the category of yogurt and cream, and from 93 to 100% in the category of fresh and frozen meat, fish and eggs. In our hypothetical reformulation scenarios, the share of products permitted for marketing to children increased substantially in most, but not all product categories examined. For example, a 10% reduction of the total sugar content of yogurt and cream results in an increase of the share of products permitted for marketing to children in this category from 73 to 93%. By contrast, even a 30% reduction in the sodium content of savoury snacks, nuts and seeds would raise the share of products permitted for marketing to children in this category only modestly from 53 to 57% (see Supplementary Table S3).

**Table 1:** Share of products meeting all nutrient and ingredient criteria of the WHO NPM (i.e. permitted for marketing to children) and of the initial^a^ and current^b^ draft of the planned Children's Food Advertising Act in Germany^3^

	Original WHO NPM (%)	WHO NPM with adaptations of initial draft (Feb 2023)^a^ (%)	WHO NPM with adaptations of current draft (June 2023)^b^ (%)
Median across product categories (IQR)	20	38	55
	(3–59)	(11–73)	(11–73)
1 Confectionery	0	0	0
2 Cakes and cookies	0	0	0
3 Savoury snacks, nuts and seeds	53	53	53
4.1 Juices	0	100	100
4.2 Dairy milk drinks	20	80	80
4.3 Plant-based milks	70	70	73
4.4 Energy drinks	0	0	0
4.5 Soft drinks, bottled water and other drinks	23	23	23
5 Ice cream	0	0	0
6 Breakfast cereals	57	57	57
7 Yogurt and cream	13	13	73
8 Cheese	20	20	20
9 Ready-made and convenience foods	60	60	60
10 Butter, other fats and oils	73	73	73
11 Bread	57	57	57
12 Pasta and grains	93	93	93
13 Fresh and frozen meat, fish and eggs	93	93	100
14 Processed meat and fish	13	13	13
15 Fresh and frozen fruit and vegetables	100	100	100
16 Processed fruit and vegetables	20	20	20
17 Savoury plant-based foods	10	10	10
18 Sauces, dips and dressings	0	0	0

Abbreviations: WHO NPM, World Health Organization Regional Office for Europe Nutrient Profile Model; IQR, interquartile range, shown here as span from the 25th to the 75th percentile.

Alt text: This table shows a comparison of product compliance with the criteria of the WHO Nutrient Profile Model and Germany's proposed Children's Food Advertising Act. The table shows the percentage of products meeting nutrient and ingredient criteria for marketing to children.

a This is the original WHO NPM with two adaptations proposed by Germany’s Federal Ministry of Food and Agriculture in the initial draft (February 2023) of the proposed law, namely a removal of the total sugar threshold for 100% fruit juice, and of the total fat threshold for milk.

b This is the original WHO NPM with adaptations proposed by Germany’s Federal Ministry of Food and Agriculture in the current draft (June 2023) of the proposed law, namely a removal of the total sugar threshold for 100% fruit juice, of the total fat threshold for milk, of the total fat threshold for plant-based milks, of the total fat and saturated fat threshold for yogurt and cream, and of the total fat threshold for fresh and frozen meat, fish and eggs.

## Discussion

Compared to the original WHO NPM, the adaptations to the ingredient and nutrient thresholds included in the current draft of Germany’s law result in a substantial increase in the median share of products permitted for marketing to children—from 20 (IQR: 3–59%) to 55% (IQR: 11–73%). In some product categories, the share of products permitted for marketing has increased markedly (e.g. from 13 to 73% for yogurt and cream, from 20 to 80% for dairy milk drinks, from 0 to 100% for juices and from 93 to 100% for fresh and frozen meat, fish and eggs). The increase in dairy milk drinks stems from the removal of the total and saturated fat threshold (the sugar thresholds for dairy and plant-based milks remains, recognizing the potential health effects associated with high sugar content in those products). The increase in the share of juices allowed for marketing to children is due to the removal of the sugar threshold for this product category. According to Germany’s Federal Ministry of Food and Agriculture, the motivation for this adaptation of the WHO NPM was that juices (that do not contain added sugars or sweeteners), while high in sugar, are also a source of vitamins which may contribute to a healthy diet.^8^ Of note, recent evidence suggests that daily consumption of 100% fruit juice is associated with a small but significant increase in body mass index in children.^9^ Overall, the substantial share of products permitted for marketing to children across most product categories indicates that claims suggesting the law would lead to a complete ban on marketing any food products are unfounded. The narrowing of the exposure definitions in the law’s current draft is likely to further limit its reach. However, according to media reports, the latest adaptations have not convinced the critics of the law within the German government, meaning that further delays and revisions limiting the law’s public health impact seem likely.

## Supplementary Material

ckae087_Supplementary_Data

## Data Availability

The data that support the findings of this study, including a full list of food items included in our analysis, are openly available and downloadable as a csv file on the Open Science Framework at https://doi.org/10.17605/OSF.IO/BJEVC.

## Other versions

An earlier version of this manuscript has been published as preprint on medRxiv at https://doi.org/10.1101/2023.11.08.23298259.^10^ The present manuscript complements an analysis of the initial proposal for the Children's Food Advertising Act from February 2023, which has been published elsewhere.^7^

Key points

- In light of evidence for adverse effects of marketing of unhealthy foods on diets and health, Germany’s Federal Ministry of Food and Agriculture announced in February 2023 a law limiting the marketing of unhealthy foods to children.
- The law’s most recent draft, dating from June 2023, uses an adapted version of the Nutrient Profile Model of the WHO Regional Office for Europe (WHO NPM) to determine which foods would be allowed to be marketed to children under the proposed law.
- This study evaluates the reach of the proposed law, showing that under the current proposal, the median share of products permitted for marketing to children is 55% across the 22 product categories covered by the law, substantially higher than the 20% permitted under the original WHO NPM.
- This analysis contributes evidence to the ongoing debate surrounding the proposed law by refuting critics' claims that the law would result in a complete and indiscriminate ban on the marketing of food products.
