# Supplementary material for: Reach and public health implications of proposed new food marketing regulation in Germany: an updated analysis
Source: Eur J Public Health. 2024 May 22;34(6):1109–11. doi: 10.1093/eurpub/ckae087 (PMC11631481; doi:10.1093/eurpub/ckae087)
Supplement: ckae087_Supplementary_Data [file ckae087_supplementary_data.pdf]

Supplementary material

Supplementary material to: Anna Leibinger, Nicole Holliday, Oliver Huizinga, Carmen Klinger, Elochukwu Okanmelu, Karin Geffert, Eva Rehfuess, Peter von Philipsborn (2024): *Reach and public health implications of proposed new food marketing regulation in Germany: an updated analysis.* Short report submitted to the European Journal of Public Health, February 2024.

Table of contents

1. Comparison of the initial and the current draft of the Children's Food Advertising Act 1

2. Nutrient and ingredient criteria of the WHO NPM and the draft Children's Food Advertising Act in Germany... 2

3. Additional analyses... 3

References... 3

1. Comparison of the initial and the current draft of the Children's Food Advertising Act

**eTable 1: Comparison of the initial (Feb. 2023) and the current draft (June 2023) of the Children's Food Advertising Act**[1-3]	
**Domain**	**Changes between the initial and the current draft**
**Nutrient Profile Model**	Removal of the total fat threshold for plant-based milks and for fresh and frozen meat, fish and eggs.<br>Removal of the total and saturated fat thresholds for yogurt and cream.
**Definition of exposure – location criteria for outdoor advertising**	Removal of playgrounds and leisure facilities from the list of areas where advertising of unhealthy foods within a 100-meter radius is prohibited
**Definition of exposure – time criteria for TV and radio advertisement**	Narrowing down of the time spans during which no advertising for unhealthy products may be aired to:<br>• Monday through Friday: 5 pm to 10 pm<br>• Saturdays: 8 am to 11 am, and 5 pm to 10 pm<br>• Sundays: 8 am to 10 pm<br><br>Besides, the time criteria no longer apply to radio advertising, but only to TV advertising.<br><br>According to the initial draft, the rule would have applied to advertising aired from 6 am to 11 pm on any day on TV or radio.

2. Nutrient and ingredient criteria of the WHO NPM and the draft Children's Food Advertising Act in Germany

The nutrient and ingredient criteria of the WHO NPM and the draft Children's Food Advertising Act in Germany are shown in eTable e2 below. Differences in the thresholds proposed by the WHO NPM and those used in the draft Children's Food Advertising Act are highlighted in yellow and explained in the footnotes.

eTable 2: Nutrient and ingredient thresholds of the WHO NPM per 100 g of product[1-4]*

Nr	Product category	Energy (kcal)	Total fat (g)	Saturated fat (g)	Total sugars (g)	Sodium (g)	Added sugars (g)	Non-sugar sweeteners (g)
1	Confectionery						0	0
2	Cakes and cookies		3			0.1	0	0
3	Savoury snacks, nuts and seeds					0.1	0	0
4	Beverages							
4.1	Juices				0**			0
4.2	Dairy milk drinks		3**				0	0
4.3	Plant-based milks		3***				0	0
4.4	Energy drinks						0	0
4.5	Soft drinks, bottled water and other drinks						0	0
5	Ice cream		3			0.1	0	0
6	Breakfast cereals		17		12.5	0.5		
7	Yogurt and cream		3***	1***	12.5	0.1		
8	Cheese		17			0.5		
9	Ready-made and convenience foods	225	17	6	12.5	0.5		
10	Butter, other fats and oils			21		0.5		
11	Bread		17		12.5	0.5		
12	Pasta and grains		17		12.5	0.5		
13	Fresh and frozen meat, fish and eggs		17***					
14	Processed meat and fish		17			0.5		
15	Fresh and frozen fruit and vegetables	Permitted						
16	Processed fruit and vegetables		3		12.5	0.5	0	
17	Savoury plant-based foods		17			0.5	0	0
18	Sauces, dips and dressings		17			0.5	0	0

Abbreviations: WHO NPM: World Health Organization Regional Office for Europe Nutrient Profile Model [4]. *The WHO NPM also includes a threshold for trans-fatty acids of 2 g/100 g of fat for all product categories, which we did not apply in our analysis as we were unable to ascertain the trans-fatty acid content of the products in our sample. **These thresholds apply only in the original WHO NPM and not in the adapted version proposed by Germany's Federal Ministry of Food Agriculture in the initial draft of the law. ***These thresholds were removed in the current draft of the proposed law from June 2023. Source: [1-4]

3. Additional analyses

Effects of specific reformulation scenarios on the share of products permitted for marketing to children are shown in eTable 3.

eTable 3: Effects of specific reformulation scenarios on the share of products permitted for marketing to children (in %)

Category	Threshold	Reformulation level				Relative increase*
		0%	10%	20%	30%	
Bread	Sodium	57%	73%	90%	90%	58%
Breakfast cereals	Sugar	57%	57%	63%	67%	17%
Ready-made and convenience foods	Sodium	60%	63%	73%	80%	33%
	Sodium and energy	60%	70%	83%	90%	50%
Savoury plant-based food	Sodium	10%	20%	23%	27%	167%
Processed meat and fish	Sodium	13%	17%	17%	17%	28%
	Total fat and sodium	13%	20%	23%	23%	77%
Savoury snacks, nuts and seeds	Sodium	53%	53%	57%	57%	7%
Yogurt and cream	Total sugar	73%	93%	93%	97%	33%
Cheese	Sodium	20%	20%	20%	20%	0%
	Total fat	20%	20%	20%	20%	0%
	Total fat and sodium	20%	20%	20%	27%	35%

*Relative increase between the baseline (no reformulation) and the -30% reformulation scenario. Note: This table shows the results for the WHO NPM with adaptations proposed by Germany's Federal Ministry of Food and Agriculture in the current draft (June 2023) of the proposed law, namely a removal of the total sugar threshold for 100% fruit juice, of the total fat threshold for milk, of the total fat threshold for plant-based milks, of the total fat and saturated fat threshold for yogurt and cream, and of the total fat threshold for fresh and frozen meat, fish and eggs.

References

1 BMEL: https://www.bmel.de/SharedDocs/Meldungen/DE/Presse/2023/230303-kinderschutz-werbung.html.

2 BMEL: https://www.bmel.de/SharedDocs/FAQs/DE/faq-lebensmittelwerbung-kinder/faq-lebensmittelwerbung-kinder_List.html Accessed April 5, 2023.

3 Referentenentwurf des Bundesministeriums für Ernährung und Landwirtschaft: Entwurf eines Gesetzes zum Schutz von Kindern vor Werbung für Lebensmittel mit hohem Zucker-, Fett- oder Salzgehalt (Kinder-Lebensmittel-Werbegesetz – KLWG) (Bearbeitungsstand: 28.06.2023): Sess. Session (2023).

4 WHO: https://www.who.int/europe/publications/i/item/WHO-EURO-2023-6894-46660-68492 Accessed March 10, 2023.
